# Levofloxacin-Induced Arthralgia in Multidrug-Resistant Tuberculosis Patients: A Case Series Spanning Three Age Groups

**DOI:** 10.7759/cureus.64955

**Published:** 2024-07-19

**Authors:** Shaily KP, Manosri Mandadi

**Affiliations:** 1 Respiratory Medicine, Dr. D. Y. Patil Medical College, Hospital & Research Centre, Dr. D. Y. Patil Vidyapeeth (Deemed to be University), Pune, IND

**Keywords:** pmdt (programmatic management of drug resistant tuberculosis), fluoroquinolones, adverse drug reaction (adr), mtb (mycobacterium tuberculosis), arthralgia, levofloxacin, mdr-tb (multi drug resistant tuberculosis)

## Abstract

Multidrug-resistant TB (MDR-TB) is a form of tubercular disease caused by a strain of *Mycobacterium tuberculosis* complex that is resistant to rifampicin and isoniazid. Microbiologically diagnosed patients are started on an all-oral longer regimen or shorter regimen based on the Guidelines on Programmatic Management of Drug Resistant TB (PMDT) in India. Fluoroquinolones (FQs), being the cornerstone in the treatment of MDR-TB, are categorized as class A drugs. Levofloxacin (Lfx) administered at a dose of 11-14 mg/kg/day holds a strong bactericidal activity against *Mycobacterium tuberculosis*. FQs are associated with a wide range of adverse drug reactions, such as nausea, bloating, headache, dizziness, and insomnia. Tendon rupture, arthralgia though rare, can also occur due to Lfx. Even though arthralgia is commonly seen in patients on Lfx-associated treatment, only a few cases have been reported in India to date.

We present a case series involving three cases of Lfx-induced arthralgia in patients of different age groups who are started on an all-oral longer regimen after they were diagnosed with MDR-TB. Based on the treatment protocol, patients were rechallenged or switched to other drugs in the replacement sequence as per the weight band.

## Introduction

Multidrug-resistant TB (MDR-TB) is a form of tuberculosis (TB) disease caused by a strain of the *Mycobacterium tuberculosis* complex that is resistant to rifampicin and isoniazid [1]. Impacting nearly 410,000 people worldwide in 2022, MDR-TB poses a significant and escalating global health threat [1]. TB is curable if diagnosed and treated promptly, making accurate and rapid detection crucial for reducing disease transmission within communities [2]. Cartridge-based nucleic acid amplification test (CBNAAT) plays a vital role in diagnosing MDR-TB [2]. This test, based on reverse transcription-polymerase chain reaction (RT-PCR), can detect both TB and rifampicin resistance (RR) within two hours [2]. Since over 90% of RR bacteria are also resistant to isoniazid, RR serves as a surrogate marker for MDR-TB [2].

Patients with no bacteriological evidence of TB but with clinical symptoms suggestive of the disease can be started on antitubercular treatment based on radiological evidence [3]. High-resolution CT (HRCT) of the thorax plays an important role in the radiological diagnosis of TB [3]. The common radiological findings of pulmonary tuberculosis include centrilobular nodules, branching linear and nodular opacities (tree-in-bud appearance), patchy or lobular areas of consolidation, and thick-walled multiple cavities [3]. Individuals diagnosed with rifampicin-resistant TB (RR-TB), isoniazid-resistant TB, and MDR-TB require regimens that include drugs such as fluoroquinolones (FQs), bedaquiline, linezolid, cycloserine, and clofazimine [4]. The regimens for MDR-TB are more expensive and cause more side effects when compared to the first-line regimens for drug-susceptible TB [4].

Accounting for a crucial component in the treatment of MDR-TB, FQs such as levofloxacin (Lfx) and moxifloxacin are categorized as class A drugs [5]. Lfx is typically administered for tuberculosis at doses of 11-14 mg/kg/day and has been well tolerated up to a dose of 20 mg/kg [5]. It holds strong bactericidal activity and involves inhibition of deoxyribonucleic acid gyrase [6]. FQs demonstrate a wide range of adverse reactions, such as nausea, bloating, headache, dizziness, insomnia, and tremors [6,7]. Though rare, musculoskeletal complications such as tendon rupture and arthralgia can occur due to Lfx; the latter is frequently treatable with symptomatic therapy [7]. Only a small number of cases of arthritis and arthralgia have been documented to date in India [8]. In this case series, we highlight a neglected adverse drug reaction (ADR) of levofloxacin-arthralgia in patients from different age groups who were started on an all-oral longer regimen for MDR-TB.

## Case presentation

Case 1

**Figure 1:**
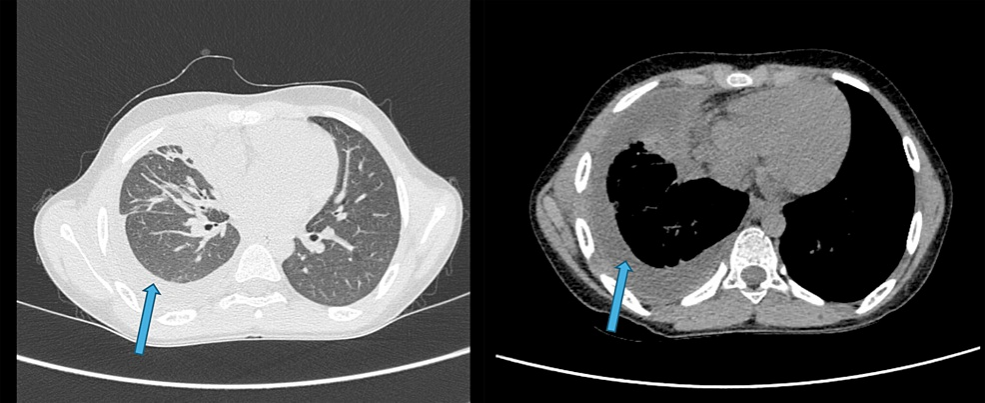
HRCT thorax - case 1 The images show moderate right-sided pleural effusion (blue arrows) with thickening of pleura and atelectatic changes in the right lower lobe and anterior segment of the right upper lobe suggestive of empyema HRCT: high-resolution computed tomography

**Figure 2:**
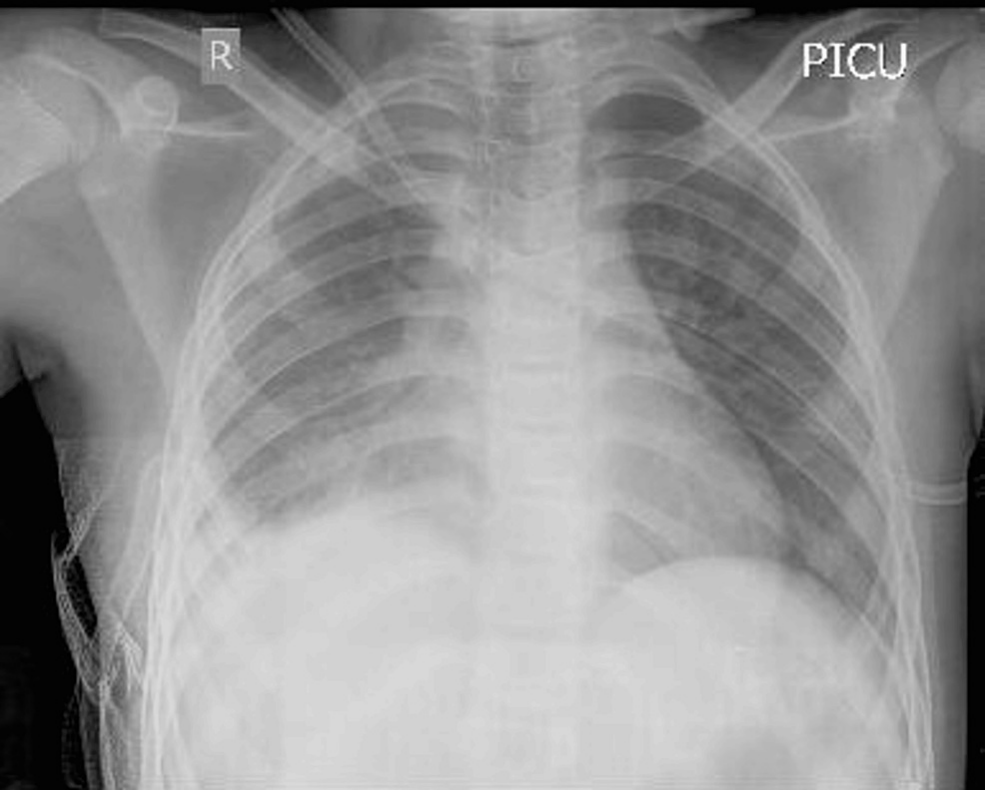
Post-decortication chest X-ray anteroposterior view - case 1 The image shows the right intercostal drainage tube in situ

A 10-year-old male presented with complaints of fever and breathlessness for two months. Chest X-ray and HRCT thorax showed right-sided moderate pleural effusion with pleural thickening and underlying atelectasis (Figure 1). Pleural fluid analysis proved to be exudative lymphocytic-predominant (Table 1). The patient underwent right-sided decortication under video-assisted thoracoscopic surgery given his empyema (Figure 2). He was microbiologically diagnosed as MDR-TB through CBNAAT and the line probe assay of the lung biopsy sample (Table 2). Based on the inclusion and exclusion criteria, he was started on an all-oral longer regimen (bedaquiline, levofloxacin, clofazimine, cycloserine, linezolid, and pyridoxine) based on his weight of 30 kg.

**Table 1:** Pleural fluid examination (indicative of exudative lymphocytic-predominant) - case 1 ADA: adenosine deaminase; LDH: lactate dehydrogenase; RBCs: red blood cells

Variable	Result	Biological reference interval
Physical		
Color	Reddish, turbid	-
Cobweb/coagulum	Absent	Absent
Deposits	Present	Absent
Chemical		
Proteins, by biuret method	1.30	Upto 3gm%
Glucose	10	>60 mg/dl
Microscopy		
RBCs	Many	Absent
Total leucocyte count	350	0-150 per cmm
Polymorphs/neutrophils	10	%
Lymphocytes	85	%
Mesothelial/macrophages	5	%
Pleomorphic cells	0	%
LDH	667 U/L	
ADA	6.03 U/L	

**Table 2:** CBNAAT and culture reports - case 1 CBNAAT: cartridge-based nucleic acid amplification test; DST: drug-sensitivity testing; MTB: Mycobacterium tuberculosis

Sample	Result
Pleural fluid culture (aerobic)	No acid-fast bacilli seen; no growth of microorganisms
Pleural fluid CBNAAT	MTB – not detected
Histopathological examination of lung tissue	Section showed fibro-collagenous tissue, granulation tissue, and many epithelioid granulomas mixed with lymphocytes and foreign body and langhans giant cells. Few granulomas showed central caseous necrosis – suggestive of likely tubercular lesion
Lung biopsy - molecular TB/DST result (line probe assay)	MTB complex detected in PCR resistant to rifampicin and isoniazid
Lung biopsy - CBNAAT	MTB high detected with rifampicin resistance
Sputum CBNAAT	MTB - not detected

One month after the initiation of the regimen, the patient reported complaints of difficulty standing from a sitting position due to severe knee pains. Further evaluation revealed normal uric acid, C-reactive protein (CRP), and erythrocyte sedimentation rate (ESR). The child was prescribed non-steroidal anti-inflammatory drugs and advised to withhold Lfx. After the resolution of symptoms, the tablet Lfx was restarted. Initially, a low dose of 500 mg Lfx was given for three days, followed by a full dose of 750 mg. During follow-up in the outpatient unit, the patient experienced a recurrence of symptoms, confirming our diagnosis of levofloxacin-induced arthralgia. Consequently, the child was switched to the next drug in the replacement sequence, pyrazinamide, based on their weight band.

Case 2

**Figure 3:**
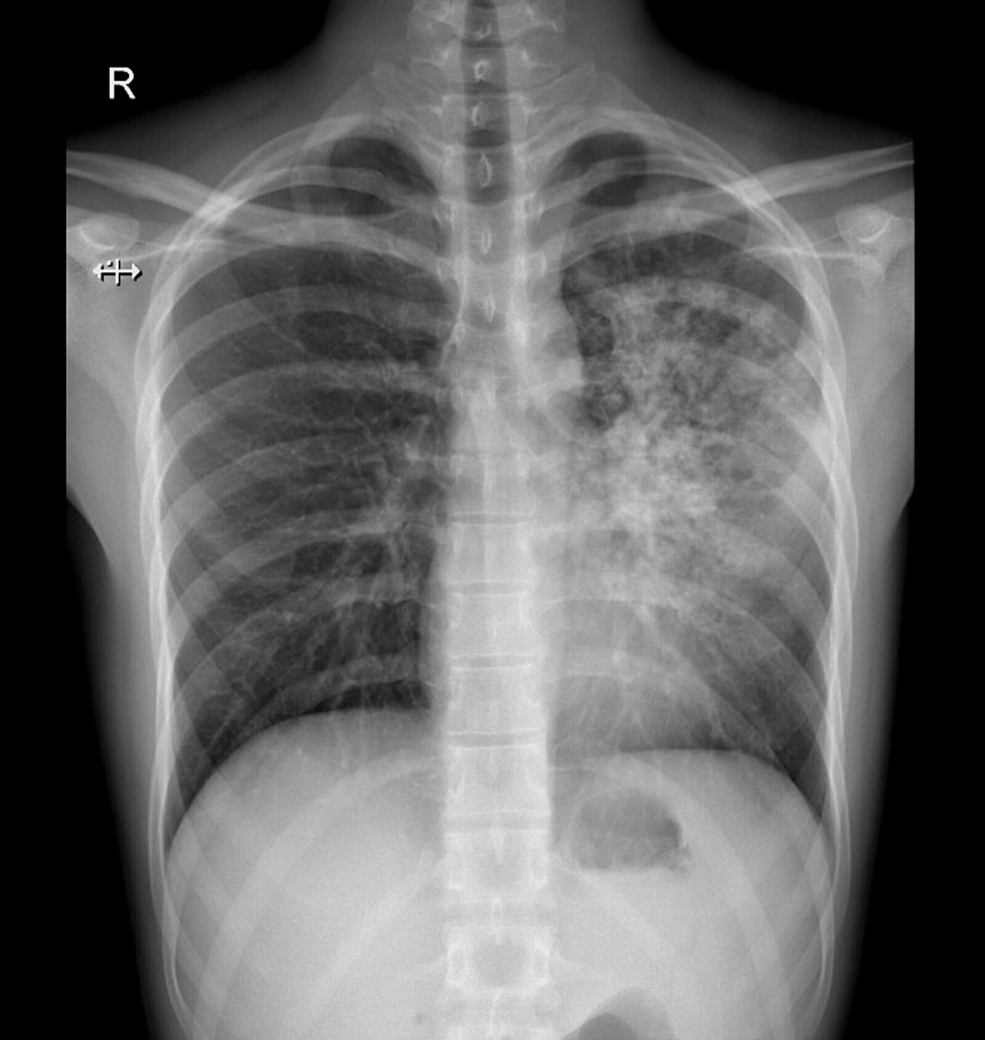
Chest X-ray posteroanterior view - case 2 The image shows diffuse heterogeneous opacities noted in the left upper and lingular lobe

**Figure 4:**
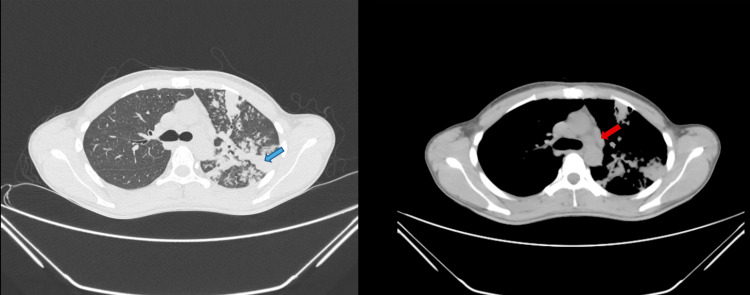
HRCT thorax - case 2 The images show a large dense consolidation with an air bronchogram in the left upper lobe (blue arrow). Mediastinal lymphadenopathy was noted (red arrow) HRCT: high-resolution computed tomography

A 33-year-old male, treated for drug-sensitive pulmonary tuberculosis one year ago, presented with complaints of fever, and cough associated with weight loss of 8 kgs in the previous two months. On further investigation, the patient’s sputum CBNAAT revealed MTB high detected with rifampicin resistance (Table 3); imaging of the chest showed large dense consolidation with air-bronchogram with multiple centrilobular nodules in the superior segment of the left lower lobe and upper lobe (Figures 3-4).

**Table 3:** Sputum studies - case 2 AFB: acid-fast bacilli; CBNAAT: cartridge-based nucleic acid amplification test; MTB: Mycobacterium tuberculosis

Sample	Result
Sputum CBNAAT	MTB high detected with rifampicin resistance
Sputum AFB - samples A and B	Positive for AFB (2+)
Sputum culture	AFB seen; no growth of microorganisms

Upon diagnosis, the patient was started on an all-oral longer regimen; 1.5 months later, he presented to our outpatient unit with joint pain involving small and large joints. During a detailed history, the patient reported stiffness in multiple joints for varying durations with no postural or diurnal variation, which was associated with restrictions to work since the last 15-20 days post-initiation of the drug-resistant TB regimen. He was admitted and worked up for arthritis - rheumatoid arthritis (RA), anti-cyclic citrullinated peptide (anti-CCP), uric acid levels, and inflammatory markers (ESR and CRP) - which were found to be normal. Meanwhile, due to high suspicion, tablet Lfx was stopped while other drugs in the regimen were continued. During this period, the patient was given a non-steroidal anti-inflammatory drug (NSAID). After a significant reduction in the symptoms, the patient was gradually rechallenged on a low dose of levofloxacin, followed by the full dose of levofloxacin reintroduced on day four of desensitization. The patient was closely monitored, and on follow-up, no resurfacing of symptoms related to arthralgia was reported.

Case 3

**Figure 5:**
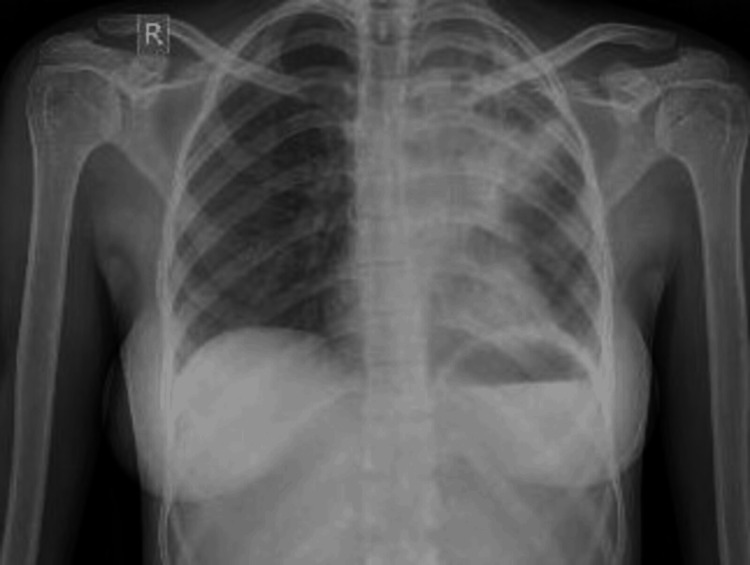
Chest X-ray posteroanterior view - case 3 The image shows diffuse homogenous opacification noted in the left upper lobe obscuring the left heart border

A 63-year-old female presented with initial symptoms of cough with expectoration and fever of one month's duration. Chest radiography of the patient showed, diffuse homogenous opacification noted in the left upper lobe obscuring the left heart border (Figure 5). Through sputum sample analysis, she was diagnosed with MDR-TB (CBNAAT - MTB high detected with rifampicin resistance) (Table 4). She was started on an all-oral longer regimen based on inclusion and exclusion criteria. She presented to us after one month of treatment initiation with a 10-day history of joint pains and complained that she was unable to carry out her daily activities.

**Table 4:** Sputum studies - case 3 AFB: acid-fast bacilli; CBNAAT: cartridge-based nucleic acid amplification test; MTB: Mycobacterium tuberculosis

Sample	Result
Sputum AFB - samples A and B	Positive for AFB (3+)
Sputum CBNAAT	MTB high detected with rifampicin resistance
Sputum culture	No growth of microorganisms

The patient was evaluated for other causes of arthritis and was asked to withhold levofloxacin as per guidelines. After one week, she was restarted on a low dose of levofloxacin of 500 mg, followed by the full dose. The patient reported a reemergence of symptoms after restarting the drug. Subsequently, a collective decision was made to switch levofloxacin to the next drug in the replacement sequence, pyrazinamide. Amikacin and delamanid were not chosen given the age and the financial factors of the patient as well as feasibility. The patient was comfortable with the new regimen and is currently being closely monitored for any new side effects.

Table 5 presents the biochemical marker tests done to rule out arthritis in all cases, while Table 6 provides a summary of all cases and their management.

**Table 5:** Biochemical marker tests done to rule out arthritis Anti-CCP: anti-cyclic citrullinated peptide; CRP: C-reactive protein; ESR: erythrocyte sedimentation rate; RA factor: rheumatoid arthritis factor

	Normal reference interval	Case 1	Case 2	Case 3
Uric acid	3.70-8 mg/dL	2.1	3.4	3
ESR	0-22 mm/hr for males; 0-29 mm/hr for females	21	10	18
CRP	<0.9 mg/dL	0.2	0.3	0.2
Anti-CCP	<5 U/mL: negative; ≥5 U/mL: positive	-	Negative (0.5)	Negative (0.6)
RA factor	<15 IU/mL	-	Negative (9.25)	Negative (9.0)

**Table 6:** Cases - description and management Lfx: levofloxacin; NSAIDs: non-steroidal anti-inflammatory drugs

Age, years	Gender	Presenting symptoms	The onset of symptoms after initiation of the regimen	Pain score (on a scale of 0-10)	Peak of symptom	Previous use of Lfx	Duration of NSAID use	Management steps taken	Reoccurrence of symptoms (after rechallenging the drug)	Outcome
10 (young)	Male	Severe knee pains, difficulty standing from sitting position	1 month	7	10 days	No	4 days	Withheld Lfx, joint rest, NSAIDs, rechallenged with Lfx	Yes	Switched to pyrazinamide
33 (middle-aged)	Male	Joint pain in multiple small and large joints, stiffness	1.5 months	6	15 days	No	4 days	Withheld Lfx, joint rest, NSAIDs, rechallenged with Lfx	No	Continued levofloxacin
63 (elderly)	Female	Joint pains, inability to carry out daily activities	1 month	6	20 days	No	4 days	Withheld Lfx, joint rest, NSAIDs, rechallenged with Lfx	Yes	Switched to pyrazinamide

## Discussion

In this case series, we discussed the cases of three MDR-TB patients of different age groups (young, middle-aged, and older) who were started on an all-oral longer regimen. In a descriptive study, Huruba et al. reported that the most common ADRs associated with FQs were arthralgia, most commonly seen with Lfx [9]. These ADRs were mostly reported between one and seven days, extending up to 30 days, and no recurrence of the symptoms was observed in the majority of the cases who were rechallenged with the drug [9].

Desensitization refers to the idea of building tolerance to a sensitized drug by administering slow increments of the drug, starting with a very small amount and gradually progressing to a full therapeutic dose [10]. Desensitization can be classified into two categories; slow drug desensitization(SDD) and rapid drug desensitization (RDD) [10]. SDD is recommended for type IV delayed hypersensitivity reaction and has shown better success rates [10]. Whereas, our patients after nearly four weeks of treatment, developed new-onset symptoms such as multiple joint pains, significantly hindering their daily activities. There were no identified alternative exposures, including other medications, to which the changes were attributable. To rule out other causes of arthralgia, these patients were worked up, and all the radiological and laboratory parameters were found to be within normal limits.

As per the Guidelines for Programmatic Management of Drug-Resistant Tuberculosis in India-2021 [11], due to the high suspicion of levofloxacin being the culprit drug, it was withheld for three to seven days. During this period, the patients were advised to have adequate joint rest and started on NSAIDs with sufficient hydration [11]. Significant symptom relief was noted by all the patients after discontinuing levofloxacin. After a symptom-free period, levofloxacin was gradually reintroduced at a low dose for three days, followed by the full dose at 11-14 mg/kg. Patients were monitored over the next month. Two of the three patients experienced the same symptoms upon rechallenging with the drug, confirming levofloxacin as the cause of arthralgia. Hence, these patients were switched to pyrazinamide according to the replacement sequence protocol.

## Conclusions

This case series discussed the occurrence of Lfx-induced arthralgia in MDR-TB patients across various age groups (pediatric, adult, and geriatric) following the initiation of an all-oral longer regimen. Lfx, a fluoroquinolone, is pivotal in MDR-TB treatment due to its potent bactericidal activity but can cause significant adverse effects, including arthralgia. The temporal relationship between drug administration and symptom onset, along with symptom recurrence upon rechallenge, confirmed the adverse reaction to the drug. These findings highlight the importance of providing comprehensive counseling to patients about the potential adverse effects of their prescribed medication regimen. Prompt decisions regarding the continuation of levofloxacin or its replacement with other drugs should be taken. Additionally, vigilant monitoring for Lfx-induced arthralgia is crucial, regardless of patient age, to enhance treatment adherence and efficacy in MDR-TB therapy.
